# Metabolomic adaptations and correlates of survival to immune checkpoint blockade

**DOI:** 10.1038/s41467-019-12361-9

**Published:** 2019-09-25

**Authors:** Haoxin Li, Kevin Bullock, Carino Gurjao, David Braun, Sachet A. Shukla, Dominick Bossé, Aly-Khan A. Lalani, Shuba Gopal, Chelsea Jin, Christine Horak, Megan Wind-Rotolo, Sabina Signoretti, David F. McDermott, Gordon J. Freeman, Eliezer M. Van Allen, Stuart L. Schreiber, F. Stephen Hodi, William R. Sellers, Levi A. Garraway, Clary B. Clish, Toni K. Choueiri, Marios Giannakis

**Affiliations:** 1 Department of Medical Oncology, Dana-Farber Cancer Institute, Boston, MA 02215 USA; 2 Broad Institute of MIT and Harvard, Cambridge, MA 02142 USA; 3 Department of Chemistry and Chemical Biology, Harvard University, Cambridge, MA 02138 USA; 4 Bristol-Myers Squibb, Princeton, NJ 08540 USA; 5 Beth Israel Deaconess Medical Center, Boston, MA 02215 USA; 6 Department of Medicine, Brigham and Women’s Hospital, Harvard Medical School, Boston, MA 02115 USA

**Keywords:** Biomarkers, Oncology

## Abstract

Despite remarkable success of immune checkpoint inhibitors, the majority of cancer patients have yet to receive durable benefits. Here, in order to investigate the metabolic alterations in response to immune checkpoint blockade, we comprehensively profile serum metabolites in advanced melanoma and renal cell carcinoma patients treated with nivolumab, an antibody against programmed cell death protein 1 (PD1). We identify serum kynurenine/tryptophan ratio increases as an adaptive resistance mechanism associated with worse overall survival. This advocates for patient stratification and metabolic monitoring in immunotherapy clinical trials including those combining PD1 blockade with indoleamine 2,3-dioxygenase/tryptophan 2,3-dioxygenase   (IDO/TDO) inhibitors.

## Abstract (web summary)

Immune-checkpoint inhibition therapy has achieved success in a subset of patients. Here the authors profiled about 200 relevant metabolites in patient serum samples from three independent immunotherapy trials and found the serum kynurenine/tryptophan ratio increases to be associated with worse overall survival.

## Introduction

Inhibition of immune-checkpoint targets including PD1 is clinically effective in a variety of cancers^1–4^. However, only a subset of patients respond and complete response remains uncommon. To understand the mechanisms of response and resistance, recent studies have focused on neoantigens^5,6^, copy-number alterations^7^, and transcriptional signatures^8,9^ of tumor tissues collected from patients treated with immune-checkpoint inhibitors. Given the known role of metabolites in modulating immunity^10^, we sought to understand how individual patients’ metabolic activities adapt to PD1 immune checkpoint blockade and how they associate with therapeutic benefits.

In the present study, by comprehensively profiling serum samples, we find prevalent kynurenine increases in both melanoma and renal cell carcinoma (RCC) patients after receiving nivolumab. Additionally, increased kynurenine metabolism correlates with worse overall survival. Our findings have implications for the design and interpretation of novel combination therapies involving checkpoint inhibition.

## Results

### Metabolomic profiling of serum samples

**Fig. 1:**
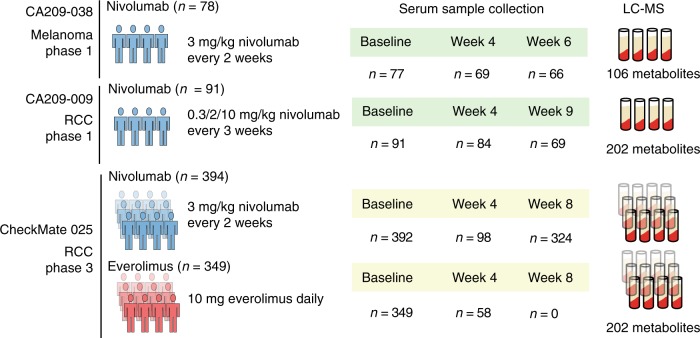
Schematic of study design and serum specimen collection. This study consisted of serum specimens from two phase 1 trials and one randomized phase 3 trial. The cancer and treatment type/dosing, number of serum samples collected at each time point, and number of metabolites identified by LC-MS are labeled as above. 63 overlapping metabolites were profiled in all three cohorts

We profiled 106–202 metabolites in pre- and multiple on-treatment patient serum samples from three independent immunotherapy trials using liquid chromatography-mass spectrometry (LC-MS) (Fig. 1, Supplementary Data 1, 2 and 3). These metabolites are involved in the metabolism of amino acids, nucleotides, nitrogen, and lipids, among others. Our study consisted of two Phase I trials (CA209-038, NCT01621490; CA209-009, NCT01358721), which included 78 patients with advanced melanoma and 91 patients with metastatic RCC treated with nivolumab^8,11^. To investigate the generalizability of our results, we also analyzed a large randomized Phase III trial (CheckMate 025, NCT01668784) with 743 RCC patients, among which 394 received nivolumab and 349 received everolimus^2^. The basic clinical and demographic features of these patients are described in Supplementary Tables 1, 2 and 3.

### Increases of kynurenine among patients receiving nivolumab

**Fig. 2:**
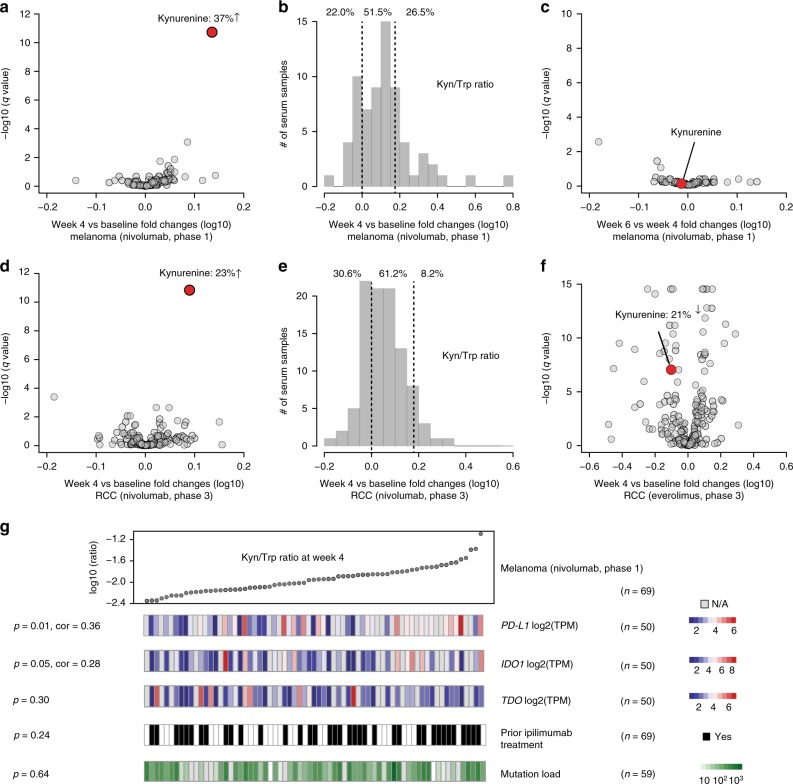
Comprehensive serum metabolomic profiling reveals significantly up-regulated kynurenine in response to nivolumab treatment. **a** Volcano plots showing average serum metabolite (*n* = 106, represented as points) level changes after 4 weeks of nivolumab treatment compared to baseline in CA209-038 melanoma patients. **b** Histogram showing the changes of kynurenine/tryptophan (Kyn/Trp) ratios 4 weeks after nivolumab treatment compared to baseline in melanoma patients. **c** Volcano plot showing average serum metabolite (*n* = 106) changes between week 6 and week 4 after nivolumab treatment in melanoma patients. **d** Volcano plots showing average serum metabolite (*n* = 202) level changes after 4 weeks of nivolumab treatment compared to baseline in CheckMate 025 RCC patients. **e** Histogram showing the changes of Kyn/Trp ratios 4 weeks after nivolumab treatment compared to baseline in CheckMate 025 RCC patients. **f** Volcano plot showing average serum metabolite (*n* = 202) level changes after 4 weeks of everolimus treatment compared to baseline in CheckMate 025 RCC patients. **g** Pearson correlation analysis between Kyn/Trp ratios and *PD-L1*/*IDO1*/*TDO* mRNA expression at week 4, prior anti-CTLA4 treatment (ipilimumab), and tumor mutation load in melanoma patients. The *q* values in **a**, **c**, **d**, **f** were calculated based on paired *t*-tests for all profiled metabolites with Benjamini-Hochberg multiple testing corrections

We first investigated the impact of nivolumab on serum metabolites during treatment compared to the baseline (prior to treatment). Among melanoma patients, kynurenine, a product of tryptophan catabolism, was the most significantly changed metabolite at week 4 and at week 6 compared to pre-treatment levels (37% and 34% increase on average, *q* < 1 × 10^−10^ and *q* < 1 × 10^−8^, respectively, paired *t*-test) (Fig. 2a, Supplementary Fig 1a). By using Kyn/Trp as a metric indicating tryptophan-kynurenine conversion, we found that this ratio falls in a range spanning approximately eight-fold among these patients, suggesting prominent individual-to-individual differences (Fig. 2b). Specifically, 78% patients had any increases and 26.5% patients had increases above 50% at week 4. On average, we did not observe further kynurenine increases between week 4 and 6 (Fig. 2c). For RCC patients treated with nivolumab in either the phase 1 or the phase 3 trials, we confirmed kynurenine as a top up-regulated metabolite following nivolumab treatment (Fig. 2d, Supplementary Fig. 1b–d). In particular, the phase 3 cohort showed 23% and 24% increase on average at week 4 and week 8, respectively, (*q* < 1 × 10^−10^ and *q* < 1 × 10^−12^, paired *t*-test). Additionally, 69.4% and 8.2% patients had Kyn/Trp increases above zero and 50% respectively at week 4 (Fig. 2e). We did not find further increases of kynurenine after week 4 (Supplementary Fig. 1e, f). Notably, patients receiving everolimus control treatment had a decrease in kynurenine (21% decrease on average, *q* < 1 × 10^−5^, paired *t*-test) (Fig. 2f) and 69% patients had reduced Kyn/Trp ratios.

Kynurenine is synthesized during tryptophan catabolism by indoleamine 2,3-dioxygenase (IDO) or tryptophan 2,3-dioxygenase (TDO), and has been shown to suppress anti-tumor immune responses^12,13^. To explore whether the circulating Kyn/Trp correlates with immune-suppression in the tumor microenvironment, we analyzed RNAseq data of tumor biopsies from the CA209-038 trial^14^. First, among all tumors sequenced, we observed on average increased transcription of *PD-L1* after nivolumab treatment among other immune-suppression regulators including *PD1*, *CTLA4*, *LAG3*, *TIM3*, and *FOXP3* (Supplementary Fig. 2a, b), suggesting up-regulation of an immune-resistance cell program at week 4. Second, we found a significant correlation between Kyn/Trp ratio and *PD-L1* expression 4 weeks after starting nivolumab treatment (Pearson correlation, *p* = 0.01; see Fig. 2g). In contrast, Kyn/Trp was not associated with prior anti-CTLA4 treatment, or tumor mutational load (Fig. 2g). Thus, the overall increase in kynurenine levels observed among patients in response to nivolumab (but not everolimus) treatment might result in an adaptive immune suppressive microenvironment counterbalancing checkpoint blockade and contribute to immunotherapy resistance independent of previously described genomic correlates^5,14^.

### Higher Kyn/Trp ratios associate with worse overall survival

**Fig. 3:**
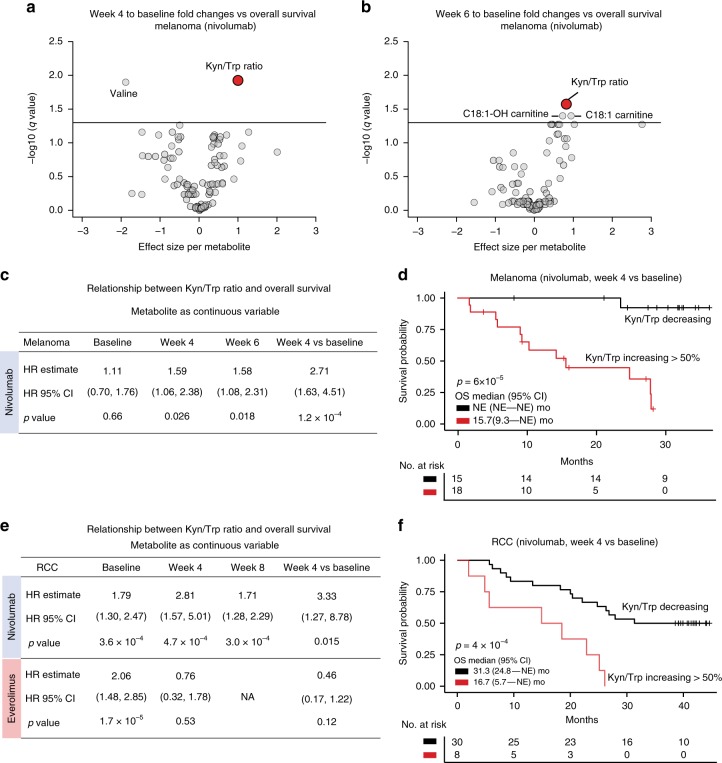
Kyn/Trp ratio alterations associate with patient overall survival in two independent cohorts. **a**, **b** Volcano plots showing associations between fold changes (log2 scale) of different serum metabolites (*n* = 106) and overall survival in melanoma patients. The effect sizes refer to the regression coefficients in a Cox proportional hazards model and the points represent different metabolites. **a** 4 weeks after nivolumab treatment versus baseline, **b** 6 weeks after nivolumab treatment versus baseline. *q* values were calculated using Benjamini-Hochberg multiple testing corrections. A cutoff at *q* = 0.05 is shown as a horizontal line. **c** Table summarizing the hazard ratios (HR) of Kyn/Trp (log2 scale) as a predictor at different time points in relation to melanoma patient overall survival using a Cox proportional hazards model. CI, confidence interval. **d** Kaplan–Meier plot comparing the overall survival in melanoma patients with >50% increases in Kyn/Trp ratios versus those with decreases. **e**, Table summarizing the hazard ratios (HR) of Kyn/Trp (log2 scale) as a predictor at different time points in relation to CheckMate 025 RCC patient overall survival using a Cox proportional hazards model. **f** Kaplan–Meier plot comparing the overall survival in RCC patients with >50% increases in Kyn/Trp ratios versus those with decreases. The *p* values in **d**, **f** were based on log-rank tests

We then sought to determine if the degrees of serum Kyn/Trp alterations correlate with clinical outcomes, focusing on overall survival (OS) for patients treated with nivolumab. Compared to all other metabolites, increases of the Kyn/Trp ratio in the melanoma cohort (week 4 or 6 vs baseline) were consistently associated with greater risks for death (*p* = 1.2 × 10^−4^, HR = 2.71, 95% CI, 1.63–4.51; *p* = 2.5 × 10^−4^, HR = 2.26, 95% CI, 1.46–3.50, respectively, Cox proportional hazards model) (Fig. 3a, b). In contrast, the baseline Kyn/Trp ratio did not significantly associate with the melanoma patients’ overall survival (*p* = 0.66, HR = 1.11, 95% CI, 0.70–1.76, Cox model) but became significant at week 4 and week 6 (Fig. 3c). This was the case even after adjusting for patient demographic characteristics and *BRAF* mutational status (Supplementary Table 4). In particular, patients with a >50% increase in Kyn/Trp had a median OS of 15.7 months while those with decreases had a median survival time of >38 months (Fig. 3d) (*p* = 6.0 × 10^−5^, log-rank test).

To confirm this result, the association between Kyn/Trp ratios and OS in a larger phase 3 trial (CheckMate 025) was evaluated using serum samples collected at different time points. We found that at baseline, higher Kyn/Trp ratios associated with shorter overall survival both for the nivolumab- and the everolimus-treated patients (*p* = 3.6 × 10^−4^, HR = 1.79, 95% CI, 1.30–2.47; *p* = 1.7 × 10^−5^, HR = 2.06, 95% CI, 1.48–2.85; Cox model) (Fig. 3e, Supplementary Fig. 3a, b). However, at week 4, Kyn/Trp significantly associated with overall survival only in the nivolumab arm (*p* = 4.7 × 10^−4^, HR = 2.81, 95% CI, 1.57–5.01; Cox model) but not in the everolimus arm (*p* = 0.53, HR = 0.76, 95% CI, 0.32–1.78; Cox model) (Fig. 3e, Supplementary Fig. 3c, d). After adjusting for patient demographic characteristics, prior anti-angiogenic treatments, and MSKCC risk group, similar results were obtained (Supplementary Table 5). For nivolumab-treated RCC patients, those with a >50% increases of Kyn/Trp had a median survival of 16.7 months while those with any Kyn/Trp decreases had a median survival of 31.3 months (*p* = 4.3 × 10^−4^, log-rank test) (Fig. 3f).

In examining the relationship between baseline Kyn/Trp and MSKCC risk scores (established clinical prognostic factors in advanced RCC)^15^, we found that patients with intermediate or poor prognosis had significantly higher baseline levels of Kyn/Trp both in the nivolumab arm (18% and 24% higher mean levels compared to the favorable group, *p* = 5.2 × 10^−9^ and 5.5 × 10^−9^, respectively, linear regression) and in the everolimus arm (10% and 23% higher compared to the favorable group, *p* = 2.3 × 10^−3^ and 7.0 × 10^−7^ respectively, linear regression) (Supplementary Table 6). However, the change of Kyn/Trp between week 4 and baseline was not associated with MSKCC risk scores in either treatment arm (*p* > 0.1, linear regression) (Supplementary Table 6). Taken together, these results suggest that while baseline Kyn/Trp is associated with disease status prior to treatment and is a prognostic marker for RCC patient survival, its dynamic alteration is independent of pre-treatment disease status.

## Discussion

In summary, we have identified increased tryptophan to kynurenine conversion in response to PD1 blockade in a subset of melanoma and RCC patients. By using two independent cohorts, we showed that Kyn/Trp temporal alterations robustly correlated with overall survival of patients receiving nivolumab. The sources that contributed to these kynurenine changes in serum are unclear. We previously profiled the kynurenine levels in 928 cancer cell lines and found marked outlier production of kynurenine and that secreted kynurenine can be attributed to *IDO* or/and *TDO* expression^16^. These data suggest that tumor cells could be a source of the kynurenine response. Here, we found a correlation between Kyn/Trp and *IDO1* but not *TDO* mRNA levels in melanoma samples 4 weeks after nivolumab treatment (Fig. 2g). However, besides tumor, other sources of host-derived tryptophan to kynurenine conversion (e.g., macrophages^17^, dendritic cells^18,19^) cannot be ruled out.

Earlier studies demonstrated that increased tryptophan to kynurenine conversion leads to inhibition of T cell proliferation^17–19^. By suppressing this pathway, tumor immune resistance could be reversed^12,13^ and checkpoint inhibition efficacy could be enhanced in animal models^20^. Our findings further illustrate that checkpoint blockade in combination with IDO/TDO inhibitors might only benefit a selected group of patients with checkpoint-inhibition-triggered kynurenine pathway activation. Given the lack of improved therapeutic outcomes with PD1 and selective IDO1 inhibition among unselected patient populations in the recent phase 3 ECHO-301/KEYNOTE-252 trial^21^, our findings highlight the need and feasibility of patient stratification by monitoring serum Kyn/Trp alterations and more generally point to the relevance of metabolic adaptations in cancer immunotherapy. Moreover, kynurenine production or kynurenine signaling may still be a relevant therapeutic target.

## Methods

### Patient population

Study design, eligibility criteria, and treatment were previously described for Bristol-Myers Squibb trials CA209-038 (NCT01621490)^11^, CA209-009 (NCT01358721)^8^, and CheckMate 025 (NCT01668784)^2^ that enrolled patients with histologically confirmed diagnoses of advanced melanoma or metastatic RCC. The patients provided written informed consent and the study protocol was approved by the Institutional Review Board of the Dana-Farber Cancer Institute/Dana-Farber/Harvard Cancer Center. The demographic and clinical characteristics of patients participated in this study are included in Supplementary Tables 1–3.

### Serum sample collection and processing

Serum was collected at the specified time-points by centrifugation at 4000 g for 4 min at 25 °C within 2 h of collection. Samples were frozen immediately and stored at or below −20 °C for up to 2 months followed by storage at −80 °C. The metabolites were profiled using liquid chromatography-mass spectrometry (LC-MS).

### Metabolomic profiling of Serum Samples from CA209-038

Positive ionization mode data were acquired using a 6495 triple quadrupole mass spectrometer coupled to a 1290 Infinity II U-HPLC system (Agilent, Santa Clara, CA). Serum samples (10 µL) were extracted using 90 µL of 74.9:24.9:0.2 (v/v/v) acetonitrile/methanol/formic acid containing stable isotope-labeled internal standards (0.2 ng/µL valine-d8, Isotec; 0.2 ng/µL phenylalanine-d8, Cambridge Isotope Laboratories). The samples were centrifuged (10 min, 9000 g, 4 °C) and the supernatants (10 µL) were injected onto a 150 × 2.1 mm Atlantis HILIC column (Waters). The column was eluted isocratically at a flow rate of 250 µL/min with 5% mobile phase A (10 mM ammonium formate and 0.1% formic acid in water) for 0.5 min followed by a linear gradient to 40% mobile phase B (acetonitrile with 0.1% formic acid) over 10 min. MS data were acquired using multiple reaction monitoring. Retention times, mass transitions, and collision energies were determined using authentic reference standards. Other MS parameters were: ion spray voltage, 3.0 kV; source temperature, 200 °C; nozzle voltage, 500 V; gas flow, 14 L/min; nebulizer, 40 psi; sheath gas, 250 °C; sheath gas flow, 1 L/min; iFunnel high pressure RF, 90; and low pressure RF, 90. Raw data were processed using MassHunter software (Agilent, Santa Clara, CA) for automated peak integration. The peak areas of 106 targeted metabolites were manually reviewed for quality of integration and compared against standard reference standards to confirm identities.

### Metabolomic profiling of Serum Samples from CA209-009 and CheckMate025

High resolution, accurate mass LC-MS data were acquired using identical sample preparation and chromatography conditions, but using a system comprised of a Shimadzu Nexera X2 U-HPLC (Shimadzu Corp.; Marlborough, MA) coupled to a Q Exactive hybrid quadrupole orbitrap mass spectrometer (Thermo Fisher Scientific; Waltham, MA). MS analyses were carried out using electrospray ionization in the positive ion mode using full scan analysis over 70–800 m/z at 70,000 resolution and 3 Hz data acquisition rate. Other MS settings were: sheath gas 40, sweep gas 2, spray voltage 3.5 kV, capillary temperature 350 °C, S-lens RF 40, heater temperature 300 °C, microscans 1, automatic gain control target 1e6, and maximum ion time 250 ms. Raw data were processed using TraceFinder software (Thermo Fisher Scientific; Waltham, MA) and Progenesis QI (Nonlinear Dynamics; Newcastle upon Tyne, UK). The identities of 202 profiled metabolites were confirmed using reference standards.

### Metabolomic data processing and quantification

Internal standards including valine-d8 and phenylalanine-d8 were used to validate consistent sample processing. In each dataset, pooled plasma reference samples were included in the analytical queue at intervals of 20 samples. To mitigate risk of temporal drift in instrument response over the course of the analyses, data for each peak (metabolite) were standardized using the ratio between the value in each sample and the nearest pooled reference multiplied by the median peak area among all pooled references. Absolute quantitation of plasma tryptophan and kynurenine was performed using external calibration curves prepared via serial dilution of stable isotope-labeled reference standards including l-tryptophan (^13^C11, 99%, Cambridge Isotope Laboratories) and l-kynurenine (D6, 97% + , Cambridge Isotope Laboratories) in human plasma.

### Statistical analysis

All statistical analyses were done in R v 3.2.1 (downloaded from https://www.r-project.org/). All statistical tests were two-sided. Statistics and relevant information including the adopted statistical tests and *p*-values are reported in the figures and associated legends. Benjamini-Hochberg multiple testing corrections were applied to obtain *q* values. For Pearson correlations, we used the *cor.test* function in R to conduct significance tests and obtain the *p*-values (two-sided). For survival analysis, we used the *survival* package in R.

### Reporting summary

Further information on research design is available in the Nature Research Reporting Summary linked to this article.

## Supplementary information

Supplementary information

Peer Review File

Description of Additional Supplementary Files

Supplementary Data 1

Supplementary Data 2

Supplementary Data 3

Reporting Summary

## Data Availability

The metabolomic data and patient information supporting the findings of this study are available in the Supplementary Data files. The raw LC-MS source data generated in this study have been deposited at www.metabolomicsworkbench.org with the project ID PR000828. The RNASeq data^14^ were downloaded from (SRA: SRP094781; BioProject: PRJNA356761).
