# Supplementary material for: Metabolomic adaptations and correlates of survival to immune checkpoint blockade
Source: Nat Commun. 2019 Sep 25;10:4346. doi: 10.1038/s41467-019-12361-9 (PMC6761178; doi:10.1038/s41467-019-12361-9)
Supplement: Supplementary file 1 — Supplementary information [file 41467_2019_12361_MOESM1_ESM.pdf]

Supplementary Information

Metabolomic adaptations and correlates of survival to immune checkpoint blockade

Li et al

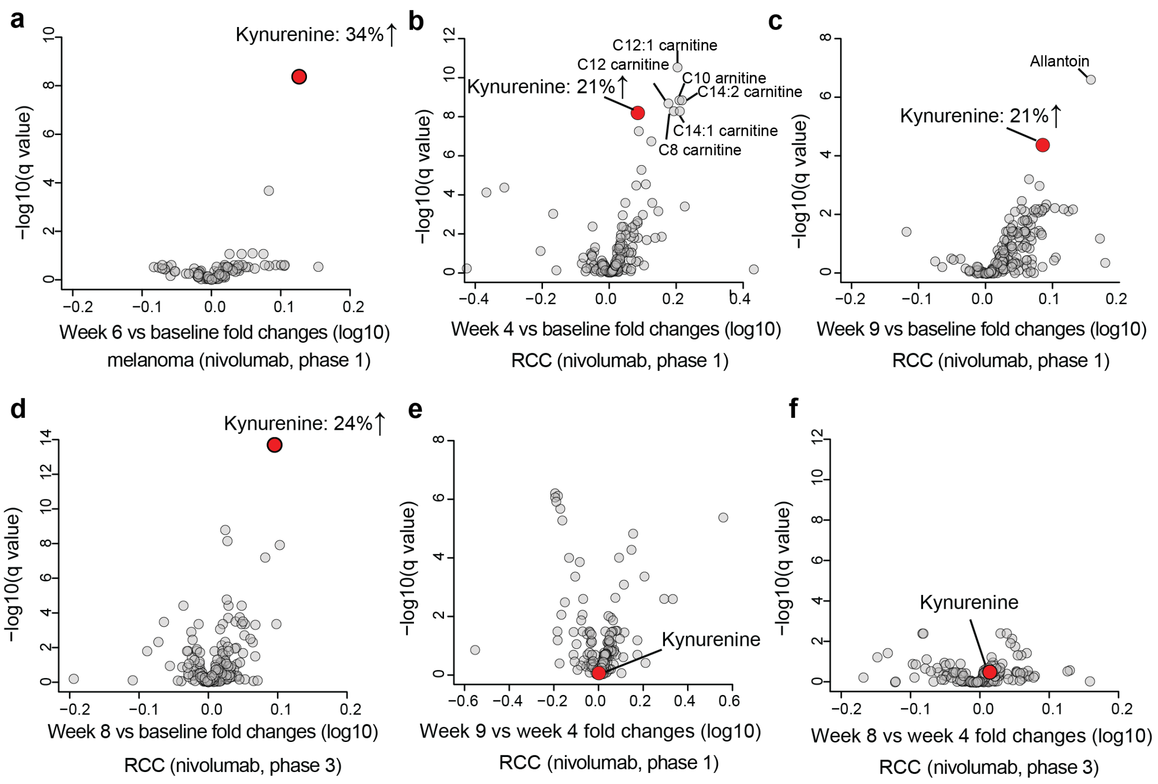

Supplementary Figure 1 | Additional information regarding kynurenine changes in serum samples.

a, Volcano plots showing average serum metabolite (n=106) level changes after 6 weeks of nivolumab treatment compared to baseline in CA209-038 melanoma patients. **b-c,** Volcano plots showing average serum metabolite (n=202) level changes after (**b**) 4 or (**c**) 9 weeks of nivolumab treatment compared to baseline in CA209-009 RCC patients. **d,** Volcano plots showing average serum metabolite (n=202) level changes after 8 weeks of nivolumab treatment compared to baseline in CheckMate 025 RCC patients. **e**, Volcano plot showing average serum metabolite (n=202) changes between week 9 and week 4 after nivolumab treatment in CA209-009 RCC patients. **f**, Volcano plot showing average serum metabolite (n=202) changes between week 8 and week 4 after nivolumab treatment in CheckMate 025 RCC patients. The q values were calculated based on paired t-tests for all profiled metabolites with Benjamini-Hochberg multiple testing corrections.

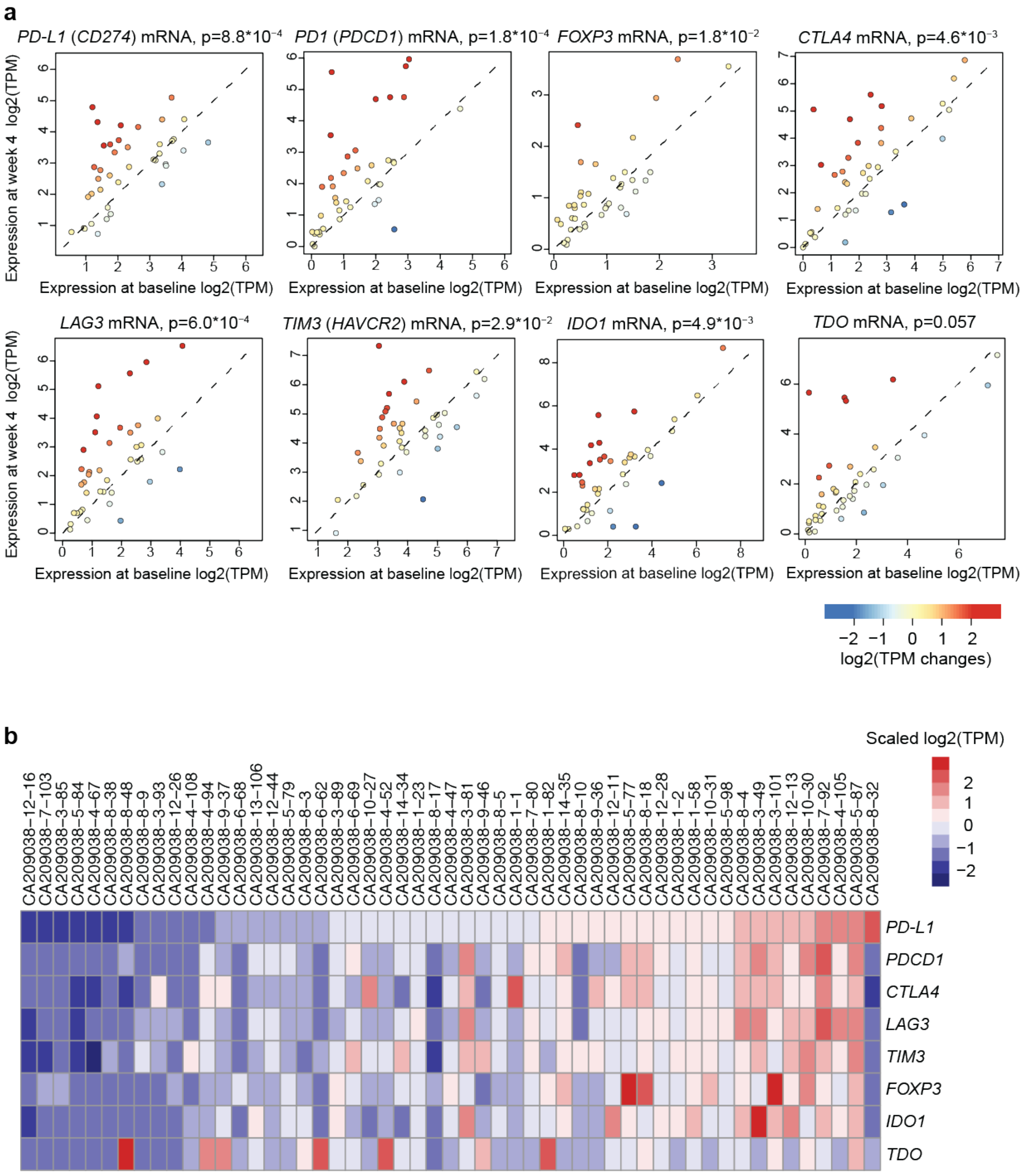

Supplementary Figure 2 | Tumor RNAseq reveals significantly up-regulated immune-suppression pathway in response to nivolumab treatment.

a, The transcription levels of negative regulators in immune responses among melanoma patients (CA209-038) at baseline and week 4. For each gene, individual patients are represented as points and are colored by the degree of alteration. The p values were calculated based on paired t-tests (n=40, paired tumor samples). **b**, The co-expression

pattern of the selected up-regulated immune-suppressive genes (y-axis) among melanoma patients (x-axis) at week 4. The TPM data have been scaled for plotting.

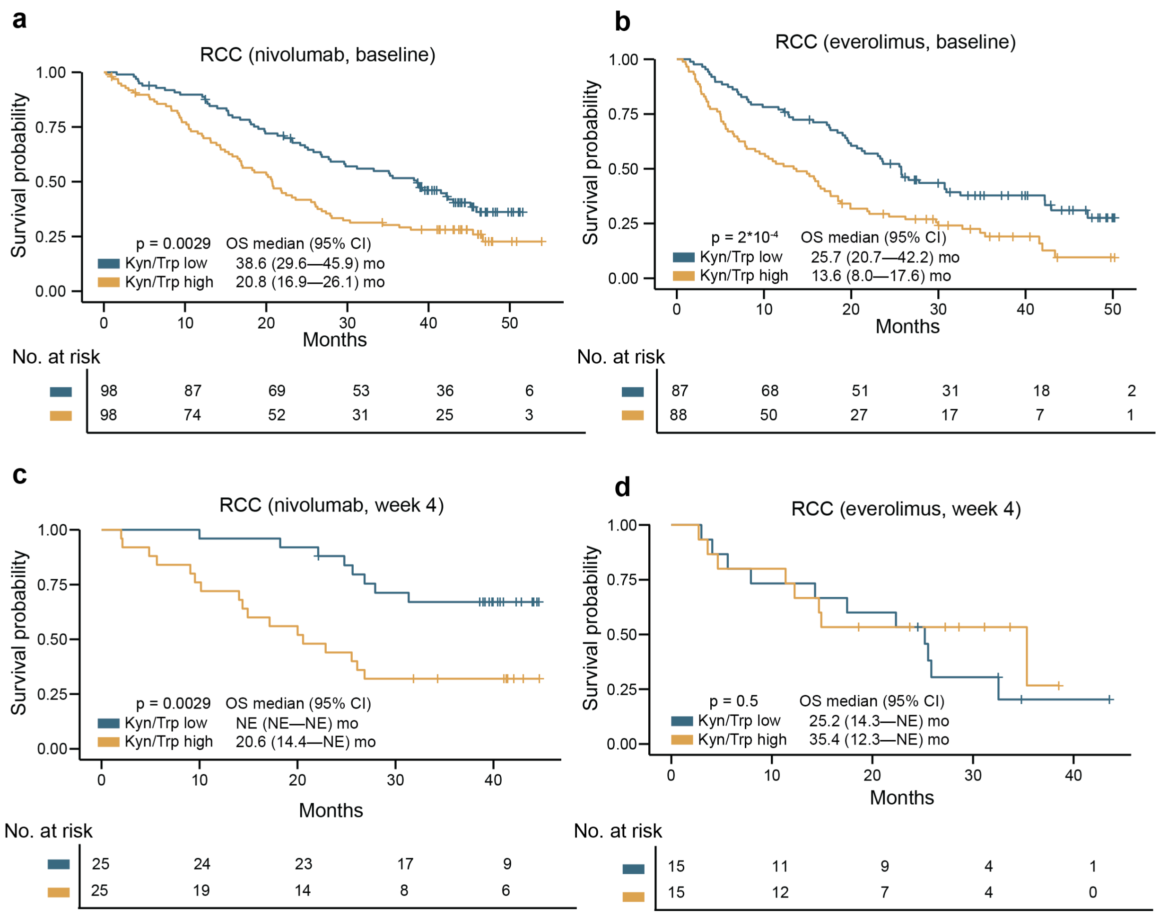

Supplementary Figure 3 | Evaluation of Kyn/Trp classification in association with CheckMate 025 RCC patient overall survival.

a-b, Kaplan-Meier plots showing overall survival in the (**a**) nivolumab-treated or (**b**) everolimus-treated RCC patients grouped by Kyn/Trp ratios at baseline. The lower quartile and the upper quartile of patients were compared. **c-d**, Kaplan-Meier plots showing overall survival in the (**c**) nivolumab-treated or (**d**) everolimus-treated RCC patients grouped by Kyn/Trp ratios at week 4. The lower quartile and the upper quartile of patients were compared. The p values were based on log-rank tests.

Characteristics	Nivolumab n=91
Sex — no. (%)	
Male	61 (67%)
Female	30 (33%)
Race — no. (%)	
White	86 (95%)
Other	5 (5%)
Age — no. (%)	
<65 yr	61 (67%)
≥65 to <75 yr	24 (26%)
≥75 yr	6 (7%)
Region — no. (%)	
U.S.	78 (86%)
Other	13 (14%)
Dose — no. (%)	
0.3 mg/kg	22 (24%)
2 mg/kg	22 (24%)
10 mg/kg	47 (52%)

Supplementary Table 1. Baseline demographic and clinical characteristics of the CA209-009 RCC patients with analyzed serum samples.

Characteristics	Nivolumab n=78
Sex — no. (%)	
Male	44 (56%)
Female	34 (44%)
Race — no. (%)	
White	77 (99%)
Other	1 (1%)
Age — no. (%)	
<65 yr	61 (78%)
≥65 to <75 yr	9 (12%)
≥75 yr	8 (10%)
Region — no. (%)	
U.S.	67 (86%)
Spain	11 (14%)
$BRAF^{V600E}$ mutation — no. (%)	
Wild type	57 (73%)
Mutant	21 (27%)

Supplementary Table 2. Baseline demographic and clinical characteristics of the CA209-038 melanoma patients with analyzed serum samples.

Characteristics	Nivolumab n=394	Everolimus n=349	Total n=743
Sex — no. (%)			
Male	300 (76%)	252 (72%)	552 (74%)
Female	94 (24%)	97 (28%)	191 (26%)
Race — no. (%)			
White	339 (86%)	308 (88%)	647 (87%)
Other	55 (14%)	41 (12%)	96 (13%)
Age — no. (%)			
<65 yr	246 (62%)	204 (58%)	450 (61%)
≥65 to <75 yr	116 (29%)	109 (31%)	225 (30%)
≥75 yr	32 (8%)	36 (10%)	68 (9%)
Region — no. (%)			
US/Canada	168 (43%)	141 (40%)	309 (42%)
Western europe	135 (34%)	120 (34%)	255 (34%)
Other	91 (23%)	88 (25%)	179 (24%)
MSKCC risk group — no. (%)			
Favorable	134 (34%)	123 (35%)	257 (35%)
Intermediate	183 (46%)	163 (47%)	346 (47%)
Poor	77 (20%)	63 (18%)	140 (19%)
Prior antiangiogenic regimens			
<2	305 (77%)	267 (77%)	572 (77%)
≥2	89 (23%)	82 (23%)	171 (23%)

Supplementary Table 3. Baseline demographic and clinical characteristics of the CheckMate 025 RCC patients with analyzed serum samples.

Relationship between Kyn/Trp and overall survival

Metabolite as continuous variable

	Melanoma	Baseline	Week 4	Week 8	Week 4 vs baseline
Nivolumab	HR estimate	1.19	1.92	1.88	3.55
	HR 95% CI	(0.70 2.01)	(1.21, 3.04)	(1.22, 2.90)	(1.79, 7.03)
	p value	0.52	0.0058	0.0045	0.00028

Supplementary Table 4. Relationship between Kyn/Trp ratios and melanoma patient overall survival adjusted by additional characteristics. This table summarizes the hazard ratios (HR) of Kyn/Trp (log2 scale) as a predictor at different time points in relation to melanoma patient overall survival. The values were calculated using a Cox proportional hazards model with additional features including sex (M/F), race (white/other), age (<65/65-75/≥75), region (U.S./other), and $BRAF^{V600E}$ status (mutant/wild type). CI, confidence interval.

Relationship between Kyn/Trp and overall survival

Metabolite as continuous variable

	RCC	Baseline	Week 4	Week 8	Week 4 vs baseline
Nivolumab	HR estimate	1.46	2.51	1.59	2.93
	HR 95% CI	(1.01 2.09)	(1.21, 5.22)	(1.16, 2.17)	(1.06, 8.13)
	p value	0.042	0.014	0.004	0.039
Everolimus	HR estimate	1.60	0.55		0.40
	HR 95% CI	(1.12, 2.28)	(0.19, 1.54)	NA	(0.11, 1.45)
	p value	0.0095	0.25		0.16

Supplementary Table 5. Relationship between Kyn/Trp ratios and CheckMate 025 RCC patient overall survival adjusted by additional characteristics. This table summarizes the hazard ratios (HR) of Kyn/Trp (log2 scale) as a predictor at different time points in relation to RCC patient overall survival. The values were calculated using a Cox proportional hazards model with additional features including sex (M/F), race (white/other), age (<65/65-75/≥75), region (North America/Western Europe/other), MSKCC risk group (favorable/intermediate/poor), and prior anti-angiogenic regimens (<2/≥2).

Linear regression of Kyn/Trp ratio (log2 scale) ~ MSKCC risk

	RCC	Nivolumab		Everolimus	
		Baseline	Week 4 vs baseline	Baseline	Week 4 vs baseline
MSKCC risk: intermediate vs favorable	coeficient	0.24	0.09	0.14	-0.11
	95% CI	(0.16, 0.32)	(-0.03, 0.22)	(0.05, 0.23)	(-0.33, 0.11)
	p value	$5.2*10^{-9}$	0.15	$2.3*10^{-3}$	0.32
MSKCC risk: poor vs favorable	coeficient	0.31	0.07	0.30	-0.06
	95% CI	(0.21, 0.41)	(-0.10, 0.24)	(0.18, 0.41)	(-0.38, 0.25)
	p value	$5.5*10^{-9}$	0.42	$7.0*10^{-7}$	0.69

Supplementary Table 6. The relationship between different MSKCC risk groups and serum Kyn/Trp ratios (log2 scale) in CheckMate 025 RCC patients.

The coefficients and p values were calculated using linear regression where MSKCC risk groups (poor/intermediate/favorable) were modeled as categorical variables.
