# Supplementary material for: Metabolomic adaptations and correlates of survival to immune checkpoint blockade
Source: Nat Commun. 2019 Sep 25;10:4346. doi: 10.1038/s41467-019-12361-9 (PMC6761178; doi:10.1038/s41467-019-12361-9)
Supplement: Supplementary file 3 — Description of Additional Supplementary Files [file 41467_2019_12361_MOESM3_ESM.pdf]

**Description of Additional Supplementary Files**

File Name: Supplementary Data 1
Description: CA209-038 melanoma patient characteristics, survival, and serum metabolomic data.

File Name: Supplementary Data 2
Description: CA209-009 RCC patient characteristics, survival, and serum metabolomic data.

File Name: Supplementary Data 3
Description: Checkmate 025 RCC patient characteristics, survival, and serum metabolomic data.
