# Supplementary material for: Metabolomic adaptations and correlates of survival to immune checkpoint blockade
Source: Nat Commun. 2019 Sep 25;10:4346. doi: 10.1038/s41467-019-12361-9 (PMC6761178; doi:10.1038/s41467-019-12361-9)
Supplement: Supplementary file 7 — Reporting Summary [file 41467_2019_12361_MOESM7_ESM.pdf]

nature research

Corresponding author(s): Toni K. Choueiri, Marios Giannakis

Last updated by author(s): Aug 6, 2019

Reporting Summary

Nature Research wishes to improve the reproducibility of the work that we publish. This form provides structure for consistency and transparency in reporting. For further information on Nature Research policies, see Authors & Referees and the Editorial Policy Checklist.

Statistics

For all statistical analyses, confirm that the following items are present in the figure legend, table legend, main text, or Methods section.

n/a	Confirmed	
☐	☒	The exact sample size (*n*) for each experimental group/condition, given as a discrete number and unit of measurement
☐	☒	A statement on whether measurements were taken from distinct samples or whether the same sample was measured repeatedly
☐	☒	The statistical test(s) used AND whether they are one- or two-sided<br>*Only common tests should be described solely by name; describe more complex techniques in the Methods section.*
☐	☒	A description of all covariates tested
☐	☒	A description of any assumptions or corrections, such as tests of normality and adjustment for multiple comparisons
☐	☒	A full description of the statistical parameters including central tendency (e.g. means) or other basic estimates (e.g. regression coefficient) AND variation (e.g. standard deviation) or associated estimates of uncertainty (e.g. confidence intervals)
☐	☒	For null hypothesis testing, the test statistic (e.g. *F*, *t*, *r*) with confidence intervals, effect sizes, degrees of freedom and *P* value noted<br>*Give P values as exact values whenever suitable.*
☒	☐	For Bayesian analysis, information on the choice of priors and Markov chain Monte Carlo settings
☒	☐	For hierarchical and complex designs, identification of the appropriate level for tests and full reporting of outcomes
☐	☒	Estimates of effect sizes (e.g. Cohen's *d*, Pearson's *r*), indicating how they were calculated

Our web collection on statistics for biologists contains articles on many of the points above.

Software and code

Policy information about availability of computer code

Data collection	Raw LC-MS data were processed using MassHunter software (Agilent, Santa Clara, CA) for automated peak integration.
Data analysis	All statistical analyses were done in R v 3.2.1 (downloaded from https://www.r-project.org/). Standard survival analysis was done with the package survival in R.

For manuscripts utilizing custom algorithms or software that are central to the research but not yet described in published literature, software must be made available to editors/reviewers. We strongly encourage code deposition in a community repository (e.g. GitHub). See the Nature Research guidelines for submitting code & software for further information.

Data

Policy information about availability of data

All manuscripts must include a data availability statement. This statement should provide the following information, where applicable:

- Accession codes, unique identifiers, or web links for publicly available datasets
- A list of figures that have associated raw data
- A description of any restrictions on data availability

The patient clinical features and metabolomic data used in the analyses have been included in the Supplementary Data.

Field-specific reporting

Please select the one below that is the best fit for your research. If you are not sure, read the appropriate sections before making your selection.

☒ Life sciences ☐ Behavioural & social sciences ☐ Ecological, evolutionary & environmental sciences

For a reference copy of the document with all sections, see nature.com/documents/nr-reporting-summary-flat.pdf

Life sciences study design

All studies must disclose on these points even when the disclosure is negative.

Sample size	The sample sizes were not pre-determined.
Data exclusions	There was no data exclusion.
Replication	Independent clinical trials were used to validate the findings.
Randomization	Study design, eligibility criteria and treatment were as previously described for Bristol-Myers Squibb trials CA209-009 (NCT01358721), CA209-038 (NCT01621490), CheckMate 025 (NCT01668784) and associated references in text.
Blinding	The investigators were not blinded to group allocation.

Reporting for specific materials, systems and methods

We require information from authors about some types of materials, experimental systems and methods used in many studies. Here, indicate whether each material, system or method listed is relevant to your study. If you are not sure if a list item applies to your research, read the appropriate section before selecting a response.

Materials & experimental systems

n/a	Involved in the study	
☒	☐	Antibodies
☒	☐	Eukaryotic cell lines
☒	☐	Palaeontology
☒	☐	Animals and other organisms
☐	☒	Human research participants
☐	☒	Clinical data

Methods

n/a	Involved in the study	
☒	☐	ChIP-seq
☒	☐	Flow cytometry
☒	☐	MRI-based neuroimaging

Human research participants

Policy information about studies involving human research participants

Population characteristics	Study design, eligibility criteria and treatment were as previously described for Bristol-Myers Squibb trials CA209-009 (NCT01358721), CA209-038 (NCT01621490), CheckMate 025 (NCT01668784) and associated references in text.
Recruitment	Study design, eligibility criteria and treatment were as previously described for Bristol-Myers Squibb trials CA209-009 (NCT01358721), CA209-038 (NCT01621490), CheckMate 025 (NCT01668784) and associated references in text.
Ethics oversight	Institutional review boards of participating institutions approved the protocol, and the study was conducted according to the Declaration of Helsinki and International Conference on Harmonisation. All patients gave written informed consent.

Note that full information on the approval of the study protocol must also be provided in the manuscript.

Clinical data

Policy information about clinical studies

All manuscripts should comply with the ICMJE guidelines for publication of clinical research and a completed CONSORT checklist must be included with all submissions.

Clinical trial registration	CA209-009 (NCT01358721), CA209-038 (NCT01621490), CheckMate 025 (NCT01668784)
Study protocol	The protocol can be assessed in CA209-009 (NCT01358721), CA209-038 (NCT01621490), and CheckMate 025 (NCT01668784) .
Data collection	This information is provided in Fig.1 and supplementary tables.
Outcomes	We used overall survival as the primary outcome. It is defined as patient death / survival at time of survey.
